# Unveiling what is absent within: illustrating anesthetic considerations in a patient with hydranencephaly – a case report

**DOI:** 10.1186/s12871-020-01142-3

**Published:** 2020-09-05

**Authors:** Alexis R. Tovar, Allison L. Thoeny

**Affiliations:** University of Arizona, 1501 N Campbell Ave, Room 4401, PO Box 245114, Tucson, AZ 85724 USA

**Keywords:** Hydranencephaly, Positioning, Airway, Anesthetic recovery, Palliative

## Abstract

**Background:**

Hydranencephaly is a rare and debilitating congenital condition in which most anesthesiologists are unfamiliar. Primary surgical treatment involves CSF diversion, though other palliative procedures requiring anesthesia are often required. With medical advancements and a resulting prolonged life expectancy, caring for these patients is becoming more routine.

**Case presentation:**

We follow an infant with hydranencephaly over three different procedures requiring anesthesia from 5 months of age to 2 years, highlighting the various anesthetic considerations.

**Conclusions:**

Anticipation of difficult positioning, deliberate airway management, and attention to anesthetic recovery were all necessary to safely care for this patient. An understanding of the challenges this particular condition poses will help anesthesiologists provide the most safe and effective care when encountering these patients.

## Background

Hydranencephaly is a rare congenital condition that occurs in less than 1 in 10,000 births in which brain development is severely restricted [1]. Cerebral structures are often limited to brainstem and thalamus only, with cerebral spinal fluid (CSF) filling a membranous sac normally inhabited by cerebral cortex [2, 3]. Diagnosis can initially be overlooked at birth due to preservation of brainstem functions, or may be confused with simple congenital hydrocephalus [3, 4]. However, within the first few weeks complications such as increased intracranial pressure (ICP), temperature dysregulation, and aspiration will arise along with a progressively enlarging head circumference [2]. As a consequence, neurological prognosis is poor and the life expectancy of these patients is reduced, with the majority dying within a few weeks or months after birth [2, 3, 5].

According to neurosurgical literature, survival in hydranencephaly past age five in the modern treatment era is becoming more common [5]. It is therefore likely that anesthesiologists will encounter such patients surviving into childhood who require anesthesia for cerebral imaging and other palliative procedures. Anesthetic considerations of the infant with hydranencephaly are complex, and pose a permanently greater challenge than hydrocephalus, due to their grave neurologic prognosis. Comprehensive management of these patients including airway, optimal anesthetic choice, safety of muscle relaxants, and postoperative monitoring has yet to be described in the anesthetic literature. We therefore delineate three separate anesthetic encounters in a patient with hydranencephaly from infancy to toddlerhood in an endeavor to augment literature on anesthetic management of such patients. Written HIPAA consent was obtained from the patient’s parent for the publication of this case report. This manuscript adheres to the applicable EQUATOR guideline.

## Case presentation

Our patient was born full-term via cesarean section. At 30 week’s gestation, a diagnosis of hydranencephaly was made with fetal ultrasound (US). At the time of delivery, his Appearance, Pulse, Grimace, Activity, and Respiration (APGAR) scores were 7 and 9 at 1 and 5 min. Primitive reflexes were intact, and physical exam was unremarkable with the exception of macrocephaly. His initial head circumference was approximately 40 cm, and his birth weight was 3.6 kg. Palliative care was consulted and helped initiate goals of care discussion and coordination of hospice care with the family. He was made “do not resuscitate” and discharged home with a poor prognosis.

Follow-up with primary care did not happen until he was 5 months of age, as the family reported difficulty with traveling given his worsening head size. They also reported progressive episodes of apnea and seizures. An MRI was ordered to evaluate for presence of cerebral tissues, and Neurosurgery was consulted for palliative shunting.

His MRI took place at 5 months of age. Head circumference measured 64 cm, and his weight was 9.5 kg. General anesthesia with a supraglottic airway device was planned as opposed to endotracheal tube (ETT) intubation due to the brevity of the procedure and intact laryngeal reflexes. Patient was placed on an MRI-compatible bed with a large shoulder roll to optimize sniffing position, given his macrocephaly. Mask induction with 8% sevoflurane followed by supraglottic airway device placement and intravenous (IV) access was uneventful. He was maintained on 2% sevoflurane. Emergence from anesthesia was performed outside the scanner to facilitate ease of access to all airway and emergency equipment. Upon removal of the airway device under low volatile concentration and presumed awake anesthetic depth, the patient went into laryngospasm. This quickly resolved with positive pressure ventilation. He was monitored for several more minutes before being transported to the post-anesthesia care unit (PACU). In PACU, he recovered without any further complications. No seizures were witnessed postoperatively, and he was discharged home.

**Fig. 1:**
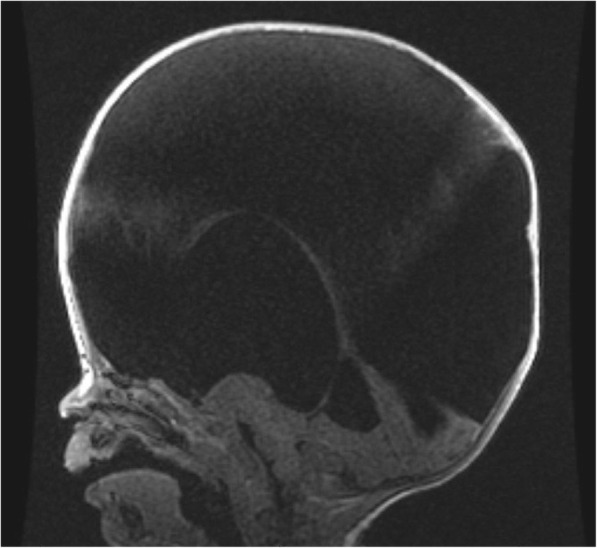
MRI sagittal view with brain parenchyma in posterior fossa, cerebral falx, thalamus, brainstem, and cerebellum visualized

**Fig. 2:**
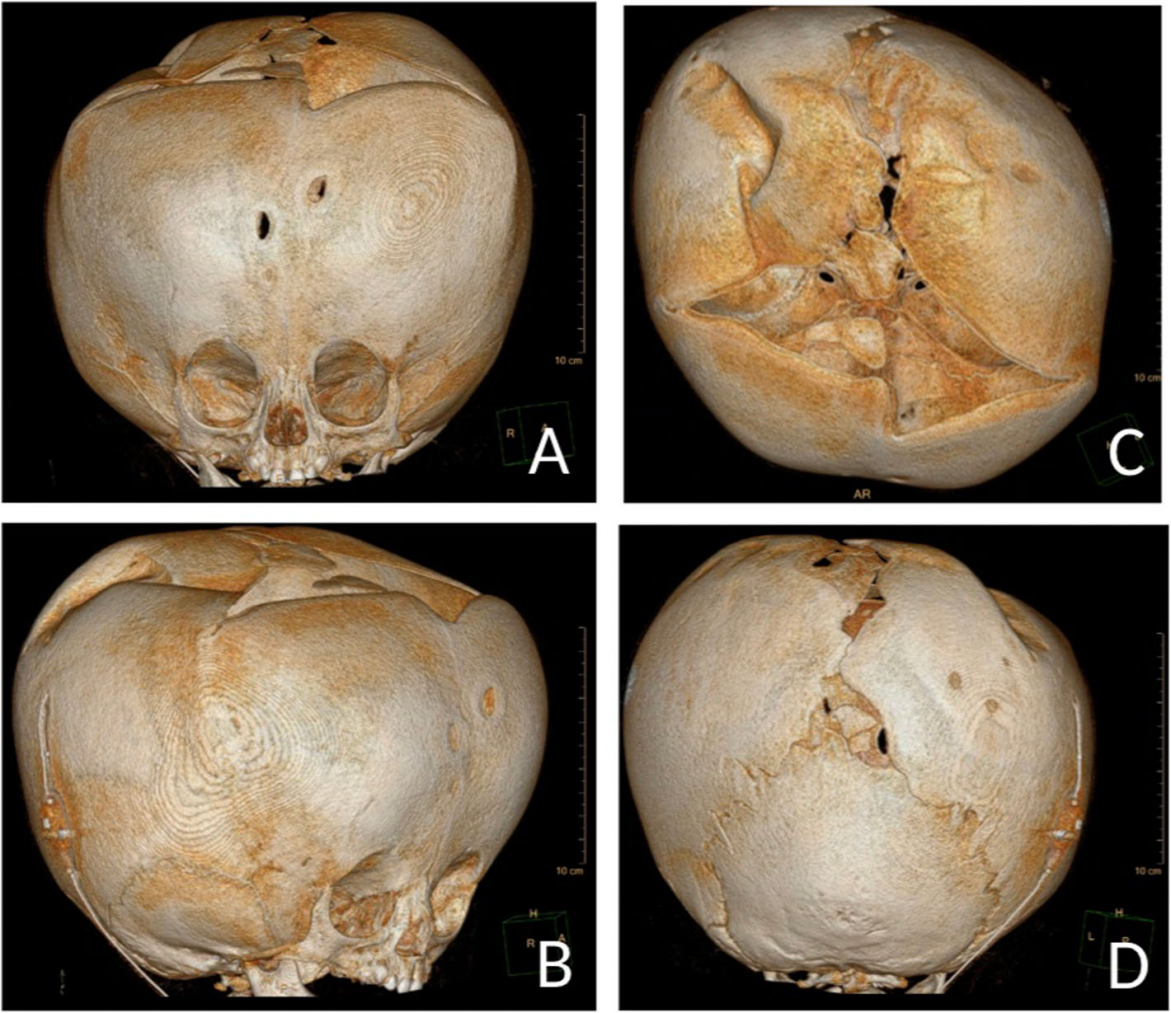
**a**-**d** 3D CT reconstruction of patient’s cranium and face after ventriculoperitoneal shunt placement

MRI imaging revealed intact brainstem and a nearly absent cerebral cortex replaced by a large membranous sac of CSF, consistent with hydranencephaly (Fig. 1). The patient underwent palliative ventriculoperitoneal shunt placement at 8 months of age at an outside hospital (Fig. 2).

**Fig. 3:**
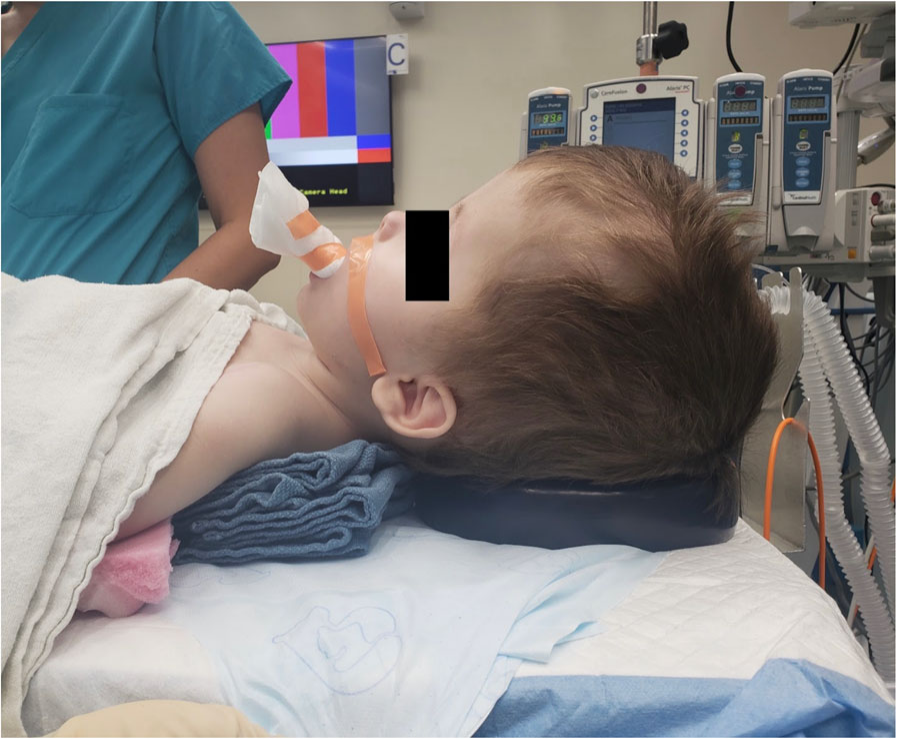
Patient with large shoulder roll and gel ring to aid in obtaining a sniffing position during induction and emergence

At 22 months of age, he returned to our institution for laparoscopic gastrostomy tube placement and management of chronic constipation secondary to opioid use as palliative medication. At presentation, he was 14.2 kg with a head circumference of 68 cm. Patient was placed supine, with blue towels underneath his shoulders to act as a shoulder roll. A gel ring was placed underneath his head for stabilization, and mask induction was performed (Fig. 3). A 24-gauge IV was obtained, he was given propofol, and his airway was secured easily using a Miller 1 blade and a 3.5 mm cuffed tube. Due to his significant baseline hypotonia, paralytic was not administered. Sevoflurane was used for maintenance of anesthesia. During the case, he also received 1.2 mg morphine for pain management, which was half of his normal home dose. Temperature was monitored with a nasopharyngeal probe, and he remained normothermic with the use of an underbody forced-air warming device and maintenance of warm ambient temperatures. Upon emergence, when noted to grimace and cry with an undetectable end-tidal volatile concentration, he was extubated. Recovery in PACU was unremarkable, and he was admitted for post-operative monitoring. His post-operative course was remarkable for opioid dependence and agitation, requiring a 24 day inpatient stay for treatment of opioid withdrawal.

Ten months later, the patient returned with stridor and labored breathing. He had several admissions prior to this for recurrent pulmonary infections, concerning for repeated aspiration events. Computed tomography (CT) imaging at this admission revealed tonsillar hypertrophy and concern for pneumonia. He was admitted to the pediatric intensive care unit (PICU) and placed on broad spectrum antibiotics for treatment of community-acquired versus aspiration pneumonia. On day 2 of admission, he underwent direct laryngoscopy and bilateral adenotonsillectomy for diagnostic and potential therapeutic treatment of his stridor. Given concern for increased airway difficulty due to his exaggerated macrocephaly, tonsillar hypertrophy, and reactive airway, spontaneous respirations were maintained during initial direct laryngoscopy with video laryngoscope using a combination of sevoflurane and IV dexmedetomidine and fentanyl. During the initial attempt at endotracheal intubation the patient went into laryngospasm with resulting hypoxia and bradycardia, which persisted despite deepening anesthetic depth and applying positive pressure ventilation. He was immediately given IV succinylcholine as well as IV atropine, with resolution of the laryngospasm. The airway was secured during the second attempt with moderate difficulty due to tonsillar hypertrophy. A 4.0 uncuffed ETT was chosen due to concerns for worsening laryngeal edema and the potential for unresolved stridor, with plans to remain intubated postoperatively. He was transported immediately back to the PICU for post-operative recovery and management, where he was successfully extubated the following day. His hospital course was complicated by returning to the operating room for tonsillar hemorrhage 9 days later, as well as for intermittent hypoxia. After 14 days in the hospital he was discharged home with oxygen.

## Discussion

Hydranencephaly represents a rare form of disturbance in cerebral development, nevertheless one increasingly encountered by anesthesiologists. The pathogenesis of the destruction of cerebral hemispheres is not well defined, but is generally considered to be due to an occlusion of the bilateral internal carotid arteries between the 8th and 12th weeks of gestation [2]. The cause for this occlusion can be variable (i.e. infectious, iatrogenic, genetic), and thus mirrors the variability seen in brain development and clinical presentation in each child [2, 3].

Prenatal diagnosis has historically been lacking, though increasingly improved with the advent of routine fetal ultrasound [2]. Approximately 1% of infants diagnosed with congenital hydrocephalus actually have hydranencephaly; differentiation between these two diagnoses is critical as congenital hydrocephalus has a significantly better prognosis [1]. However, with the quality of medical care and surgical interventions now available, life expectancy in hydranencephaly can extend once a child survives past their first 2 years of life [6].

Treatment in this population is non-curative and involves ethical and medical considerations of what course may be optimal for each child, as the degree of neurologic impairment is not improved with surgical intervention. Surgical interventions, namely CSF diversion procedures, are being performed around the world however with success in improving quality and duration of life in hydranencephaly [5, 7]. Anesthesiologists are thus tasked with providing care beyond initial palliative shunting, which may include anything from shunt revisions to enteral feeding access.

A careful and deliberate approach to anesthetic care of these rare and poorly affected patients is a daunting task for any anesthesiologist as management is largely unknown. Apart from one report we found demonstrating feasible ketamine use in an infant with hydranencephaly in 1974 [8], the literature is void of anesthetic management in hydranencephaly. Given the irreversible extent of neurologic disturbance in these infants, we believe the considerations for anesthesia in patients with hydranencephaly are in fact complex, and as seen in our patient, may include anticipation of difficult positioning, airway management, optimal anesthetic technique, and anesthetic recovery. Maintaining appropriate hemodynamics and normothermia are also essential as complications from increased intracranial pressure, such as aspiration and seizures, can occur [2].

With any exaggerated macrocephaly, as previously discussed in management of infants with hydrocephalus [9], it is vital to allow proper extension of the head in sniffing position by placing a large shoulder roll and stabilizing the head with a small towel or ring. In anticipation of a difficult airway, maintaining spontaneous ventilation is beneficial. We believe that using sevoflurane for mask induction may be advantageous, given the possible decreased intracranial compliance. With the anticipation of a difficult airway, the use of a video laryngoscope as the initial form of laryngoscopy or as an alternative can also be considered.

If IV induction is feasible and the airway appears favorable, rapid sequence intubation may be considered given the higher aspiration risk to these patients. A non-depolarizing agent is arguably best for paralysis as succinylcholine can result in profound bradycardia in infants with autonomic dysfunction, which is a presumed expectation in this population. Alternatively, if succinylcholine is required for ideal intubating conditions or as an emergency rescue medication, as was the case in our patient for resolution of laryngospasm, one should administer atropine concurrently. Consideration of the risk-benefit balance between aspiration risk and neuromuscular recovery in each patient context is prudent, however. This may lead the clinician to avoid use of neuromuscular relaxants all together, as was the case for our patient during his gastrostomy tube placement.

Maintenance of anesthesia may be accomplished with volatile or IV anesthetics, as an optimal agent for patients with hydranencephaly has not been identified in literature. Though maintenance of cerebral perfusion pressure (CPP) may not be as critical in these children due to lack of cerebral structures, an agent that decreases ICP and maintains hemodynamics is still beneficial to avoid seizures, aspiration, and maintain cardiac output to other vital organs.

Our patient tolerated sevoflurane anesthesia over three different procedures very well. An alternative for shorter procedural sedation may be ketamine. Although ketamine is a relative contraindication with increased ICP due to its ability to increase cerebral metabolic rate, it does preserve cerebral blood flow and mean arterial pressure. In fact, a clinical trial of 30 intensive care pediatric patients receiving ketamine as procedural sedation or as therapy for intracranial hypertension demonstrated an increased CPP and decreased ICP by 30% [10]. Ketamine has been used as a successful alternative in the past in an infant with hydranencephaly [8].

Another challenge is that assessment of recovery from anesthesia may prove difficult, given limited cognitive function and general hypotonia [3]. It is critical to completely reverse any residual paralysis in order to return the patient to baseline neuromuscular tone. Patients with hydranencephaly are at risk for respiratory distress and prolonged mechanical ventilation [5]. If benzodiazepines or narcotics are required, short-acting agents should be chosen to facilitate a rapid recovery and avoid post anesthetic apneic events. To avoid airway complications during emergence as seen in our patient, adequate time should be given for elimination of anesthetic effects and stable respiratory mechanics observed before extubation is attempted.

There are limitations to our observations seen in this case report that merit acknowledgement. Principally, we practice at an academic institution in which a different attending and resident anesthesiologist cared for our patient at each anesthetic encounter, reflecting the variability in anesthetic technique and management between encounters. This further emphasizes the importance of publishing collective observations and recommendations to guide future anesthetic considerations of hydranencephaly. Furthermore, since we did not employ IV drugs such as propofol for maintenance of anesthesia, we are unable to comment if that might constitute a good choice. Lastly, we understand that further study of other children with hydranencephaly is indicated to draw formal conclusions about anesthetic management.

Patients with hydranencephaly are a rare population to encounter, even for a pediatric anesthesiologist. However, with advancing medical capabilities and extended life expectancy, these encounters are becoming more common. Though similar to congenital hydrocephalus in terms of airway management and difficulty with positioning, patients with hydranencephaly distinguish themselves by being a continuing anesthetic challenge throughout their life; this is in part due to their lack of neurological recovery after CSF diversion procedures. With keen preparation and consideration of difficult positioning, airway, and anesthetic recovery, anesthesiologists can provide a safe and efficacious anesthetic for the variety of procedures that these children may require.

## Data Availability

Not applicable.

## Abbreviations

APGAR: Appearance, pulse, grimace, activity, and respiration
CPP: Cerebral perfusion pressure
CSF: Cerebrospinal fluid
CT: Computed tomography
ETT: Endotracheal tube
ICP: Intracranial pressure
IV: Intravenous
MRI: Magnetic resonance imaging
PACU: Post anesthesia care unit
PICU: Pediatric intensive care unit
US: Ultrasound
